# The Effects of Reduction in Food Intake on Growth and Differentiation of a Squamous-Celled Carcinoma in Mice, with Special Reference to Experimental Chemotherapy

**DOI:** 10.1038/bjc.1957.55

**Published:** 1957-09

**Authors:** A. E. G. Pearson

## Abstract

**Images:**


### THE EFFECTS OF REDUCTION IN FOOD INTAKE ON GROWTH AND DIFFERENTIATION OF A SQUAMOUS-CELLED CARCINOMA IN MICE, WITH SPECIAL REFERENCE TO EXPERIMENTAL CHEMOTHERAPY

A. E. G. PEARSON

*From the Department of Experimental Pathology, Mount Vernon Hospital, Northwood, Middlesex*

Received for publication May 7, 1957

THE inhibitory effect of a reduction in body weight on tumour genesis and growth in experimental animals has been studied for some years. A reduction in caloric intake has been shown to inhibit the growth of Sarcoma 180 in mice (Bischoff and Long, 1938). Variations in the constituents of the diet have shown that a reduction in fat or protein has no inhibitory effect, and variations in vitamin content have produced a diversity of results with few instances of any inhibitory effects. This literature has been comprehensively summarised by Tannenbaum and Silverstone (1953). Complete regressions were rare (Tannenbaum, 1940) and Tannenbaum and Silverstone (1953) stated that underfeeding or caloric restriction did not appear to be a successful means of controlling the growth of tumours. Barvick and Goodson (1954) showed that food restriction for 24 to 48 hour periods produced no significant inhibition of Sarcoma 180, and in mice on total starvation the tumour sizes at death (5 days) were one-third that of the controls.

The present study, with a squamous-celled carcinoma in mice, shows a high proportion of regressions resulting from underfeeding, and relates the effect to the degree of differentiation of the tumour.

### MATERIALS AND METHODS

The tumour used in this study was a homologous squamous-celled carcinoma in RIII strain mice. Sarcoma 37 in RIII strain mice was also used in a few parallel experiments. The mice used in each experiment were inoculated subcutaneously in the right flank with portions about 1 mm$^3$. taken from the cortex of one parent tumour. The transplants became measurable (about 18 mm.$^2$) about seven days after implantation, and were then divided into two groups—each group containing at least ten mice. The control group received water and diet 41* *ad lib.*, the treated group received water *ad lib.* and a proportion of the estimated normal consumption of diet 41. It was found that 2 g. per day for each female and 3 g. per day for each male mouse resulted in a loss in body weight of from 20 to 25 per cent. This degree of starvation was the maximum attempted in this series of experiments. The mice in both groups were kept four or five to a cage ; the individual weight variations were found to be consistent throughout the groups, consequently no attempt was made to use individual cages.

* The percentage composition of diet 41 (Bruce, 1950) is as follows : Wholemeal flour 46, ground oats 40, white fish meal 8, dried yeast 1, dried skimmed milk 3, cod-liver oil 1, sodium chloride 1.

The tumours were measured by calipers and the area taken as the product of the major and minor axes. This value serves for comparing tumour growth rates, but does not represent absolute tumour size. All tumours used in an experiment were fixed in Susa, and stained with haematoxylin and eosin. The control tumours were fixed when the average size had reached about 120 mm$^2$.; the tumours from mice on a restricted diet were fixed after about twenty-five days.

### RESULTS

Fig. 1 illustrates the tumour sizes in a typical experiment containing twenty mice in each group. Fig. 2 represents the average tumour sizes related to body weight changes in control and treated groups in another experiment also containing twenty mice in each group. In Fig. 1 it can be seen that six tumours

FIG. 1.—The effect of a reduced diet intake producing a body weight loss of 22 per cent, on individual tumour sizes of a squamous-celled carcinoma in female RIII strain mice.

* Mice died from Salmonella infection.

in the treated group regressed completely, and histological examination failed to show the presence of any malignant cells. The tumours from the treated group at twenty-five days showed very small areas of malignant tissue, the bulk consisting of keratin pearls and differentiating cells. Typical proportions of malignant tissue in control and treated tumours of comparable size are illustrated in Fig. 4 and 5. In the experiment represented in Fig. 2, four tumours regressed and three others were regressing and consisted almost entirely of keratin pearls with malignant tissue areas reduced to a very small proportion of the whole.

Spontaneous regressions in this tumour occur at an incidence of about 4 per cent. In experiments in which the food intake was reduced to a lesser extent the degree of inhibition varied with the body weight loss (Fig. 3).

The histological examination of this tumour indicated that one effect associated with a reduction in diet intake was an increase in the degree of differentiation. In typical control tumours of about 80 mm.$^2$ in area, actively dividing tissue was found in most parts of the tumour, with areas of keratin pearl formation restricted

FIG. 2.—The effect of a reduced diet intake on the average size of a squamous-celled carcinoma in male RIII strain mice.

to certain regions (Fig. 4). Postmitotic cells which differentiated to form the keratin pearls were not usually found in the malignant regions of the tumour. The differentiating cells were recognised by an increase in the proportion of cytoplasm to nucleus, and a decrease in the basophilia of the cytoplasm. In tumours growing in mice on a restricted diet the malignant regions were confined almost entirely to the periphery of the tumour (Fig. 5).

### DISCUSSION

It is suggested that the maintenance of the malignant condition of the cells of this tumour depended on the concentration of a substance or substances in the blood. If this concentration is reduced below a certain level the mitotic malignant cells pass into a postmitotic differentiating condition. The presence of differentiating cells in certain regions of control tumours and the restriction of mitotic cells to the periphery of treated tumours suggests local variations in the vascularity of the tumours. Experiments have been conducted to determine the effects of underfeeding on the establishment of transplants of this tumour. Preliminary results indicated that the establishment and early growth were not affected ; these experiments are to be published in greater detail.

Parallel experiments on a heterologous undifferentiated tumour—Sarcoma 37—have shown that a significant inhibition was produced by a reduction in food intake, but no regressions occurred and a resumption of normal growth

FIG. 3.—The relationships between tumour growth inhibition of a squamous-celled carcinoma in RIII strain mice and loss in body weight, under conditions of a reduced diet intake and in chemotherapeutic trials of benzoqinone-dioxime and other compounds.

rate occurred rapidly on the resumption of *ad lib.* feeding. No histological differences between tumours of wholly or partially fed animals have been found in these experiments.

*Implications for experimental chemotherapy*

In the course of chemotherapeutic trials in which the drug under consideration was incorporated in the normal diet, a loss in body weight of the treated animals has been frequently encountered. This weight loss has been related to a reduced diet intake due either to the unpalatability of the drug or its general toxic effect. The inhibitory effect of this weight loss on tumour growth was usually sufficient to mask any selective inhibitory action by the drug. If sufficient data were available for any tumour relating the degree of inhibition to proportionate weight loss, then it might be possible to detect a selective inhibitory effect by the drug.

Fig. 3 represents the relationships between weight loss and inhibition for the squamous-celled carcinoma used in this study, under conditions of partial starvation and in chemotherapeutic trials. It can be seen that the results from the benzoquinone-dioxime trials were consistent and that the degree of inhibition was greater than with animals on a reduced diet for a similar degree of weight loss. Animals fed with other compounds and tested on this tumour showed a relationship between weight loss and inhibition similar to that for animals on a restricted diet intake. In Fig. 3 the inhibition is calculated as a percentage difference, between control and treated groups, of the increase in tumour size over the period of the experiment, expressed in terms of average tumour area. The percentage loss in weight took into account any variations in the average control weights, and was calculated as a percentage of the average starting weights of the two groups ; in this way it was found that the calculated weight loss was consistent for either male or female mice.

### SUMMARY

(1) The effects of a reduction in diet intake on the growth of a squamous-celled carcinoma have been studied.

(2) A substantial inhibitory effect with a high proportion of complete regressions was observed.

(3) The marked inhibitory effect is related to an increase in the degree of differentiation of the tumour.

(4) The consistent relationship between the degree of body weight loss and tumour inhibition is discussed in connection with experimental chemotherapy.

My thanks are due to Mr. F. W. Butcher for assistance with the animal experiments and to Mr. D. Astwood for assistance with the chemical preparations.

The expenses of this research were defrayed from a block grant by the British Empire Cancer Campaign.

### REFERENCES

BARVIK, L. AND GOODSON, L. H.—(1954) *J. nat. Cancer Inst.*, **15**, 177.
BISCHOFF, F. AND LONG, M. L.—(1938) *Amer. J. Cancer*, **32**, 418.
BRUCE, H. M.—(1950) *J. Hyg., Camb.*, **48**, 171.
TANNENBAUM, A.—(1940) *Amer. J. Cancer*, **38**, 335.
*Idem* AND SILVERSTONE, H.—(1953) *Advanc. Cancer Res.*, **1**, 452. New York (Academic Press Inc.).

---

### EXPLANATION OF PLATE

Fixed in Susa fixative and stained with haematoxylin and eosin.

FIG. 4.—Section through centre of control tumour. × 8.

FIG. 5.—Section through centre of tumour in mouse on reduced diet intake. × 10.

FIG. 6.—Cortical region of control tumour in an area of differentiation and keratin pearl formation. × 80.

FIG. 7.—Cortical region of tumour under the influence of reduced diet intake in an area where viable cells are present ; showing extensive differentiation and formation of keratin pearls. × 80.


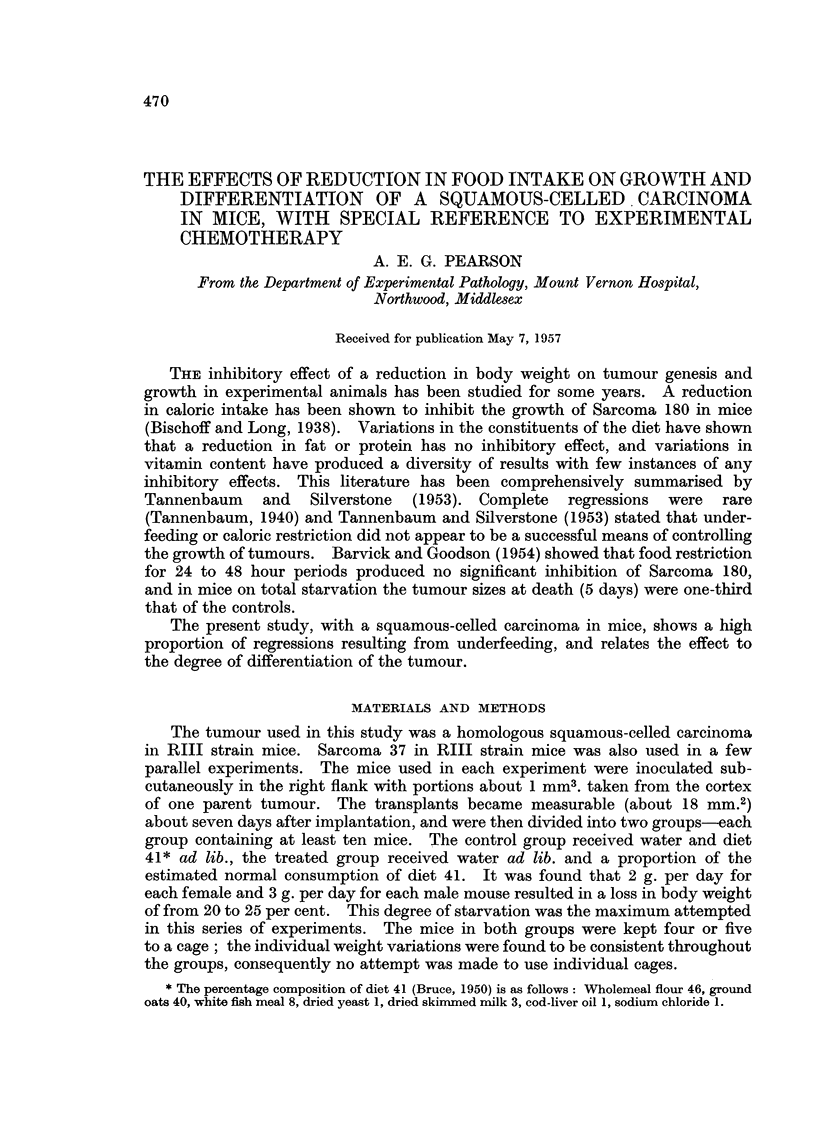

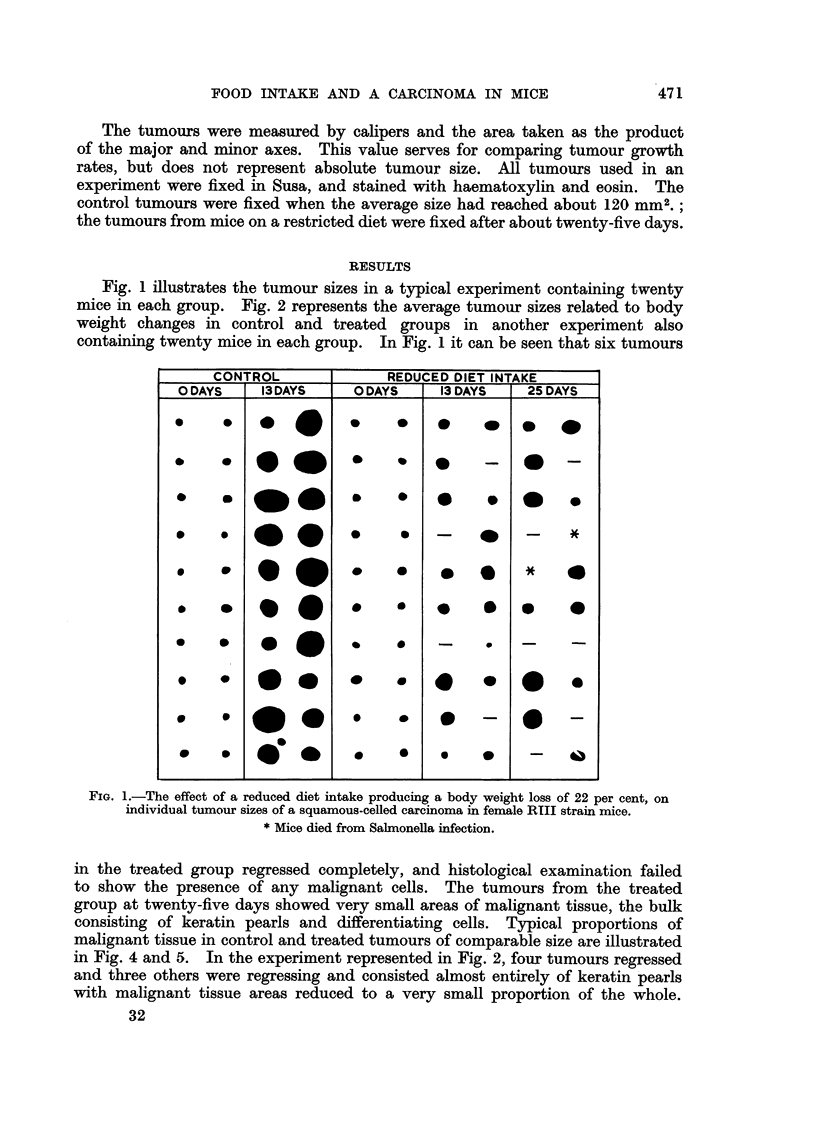

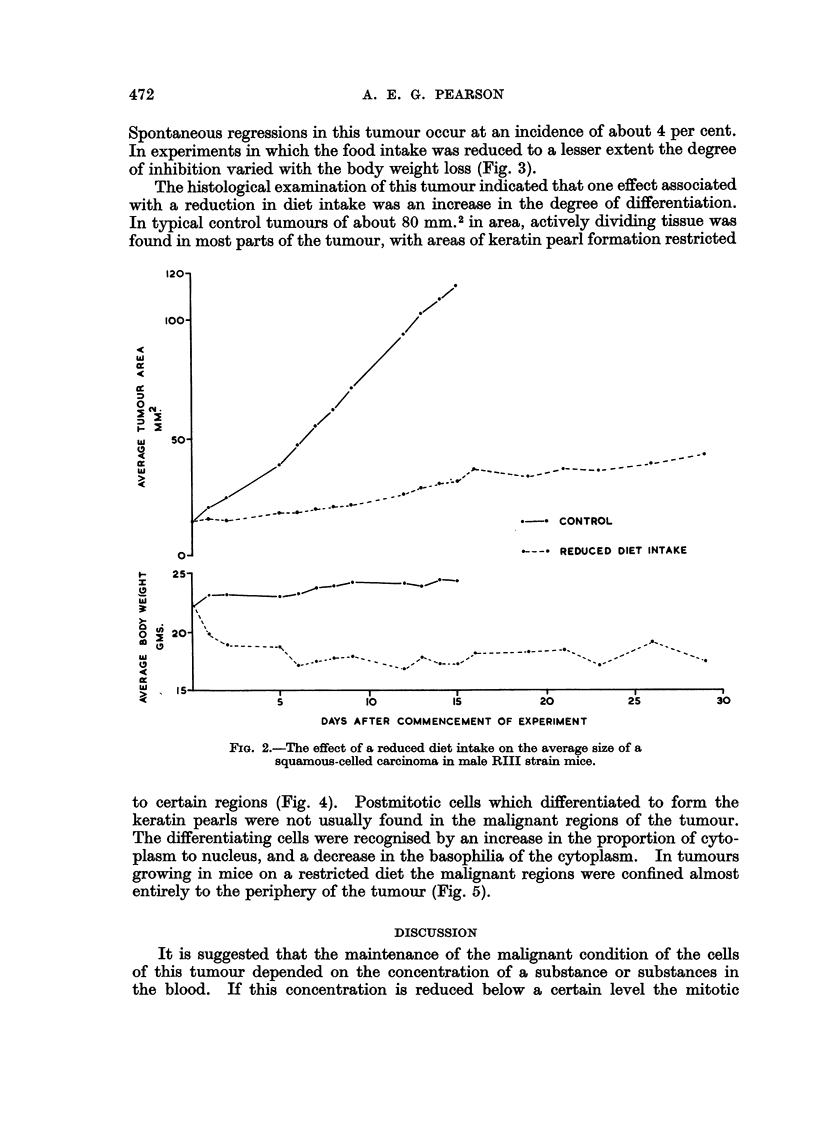

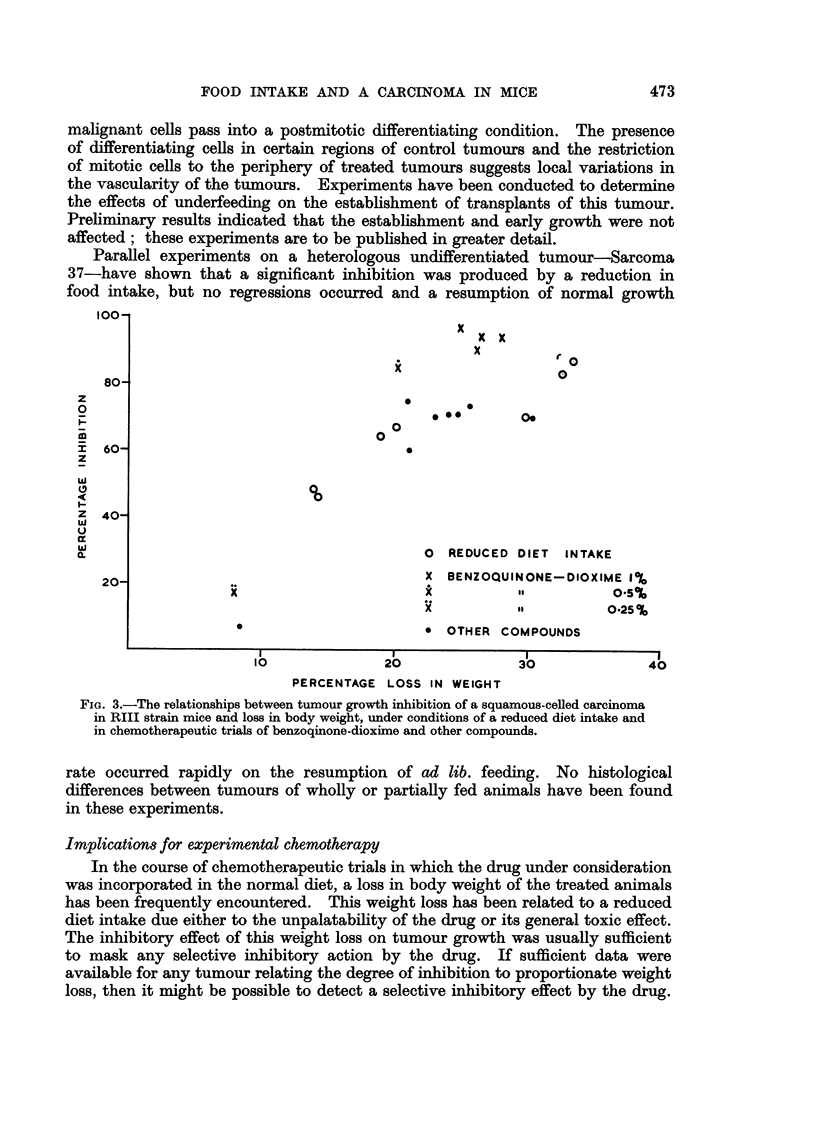

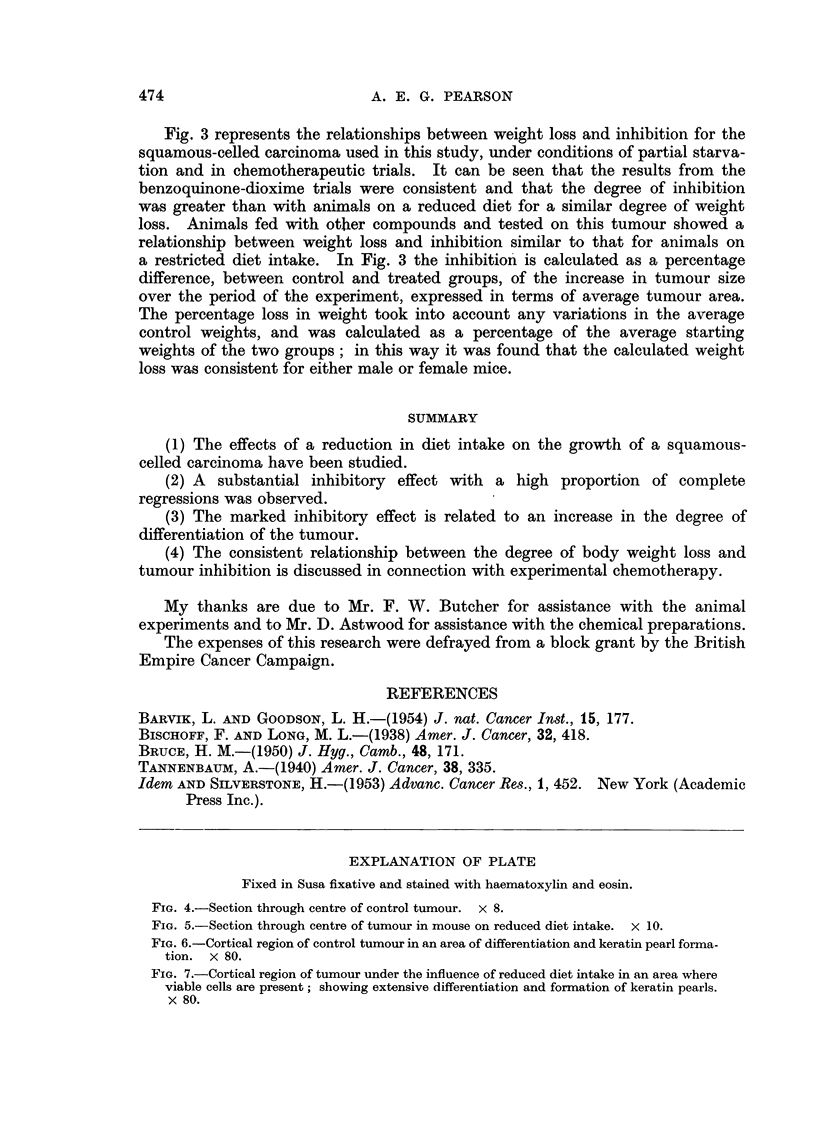

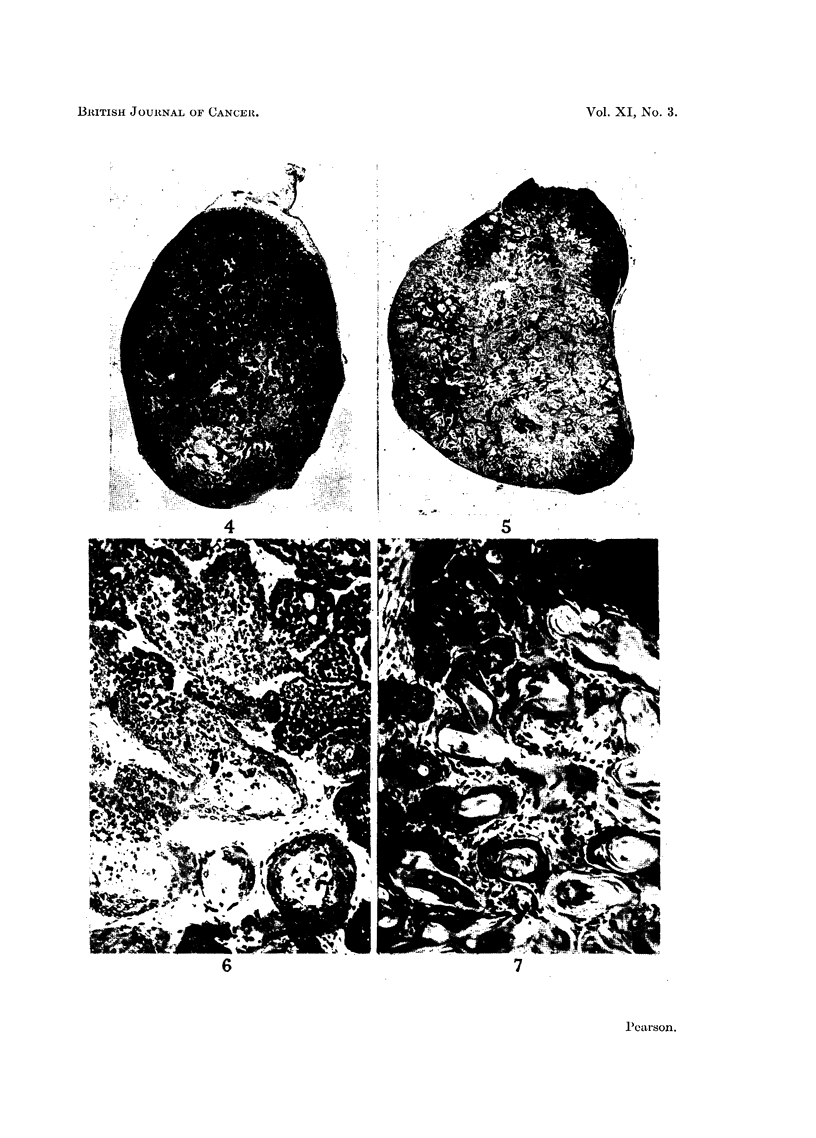


## References

[OCR_00363] BARVICK L., GOODSON L. H. (1954). Effects of combined chemotherapy on sarcoma 180, with special reference to food intake, body-weight changes, and survival time.. J Natl Cancer Inst.

